# NUDT21 relieves sevoflurane-induced neurological damage in rats by down-regulating LIMK2

**DOI:** 10.1515/biol-2022-0486

**Published:** 2023-04-15

**Authors:** Yuanping Zhong, Pengcheng Zhao, Chao Zhang, Zhenyu Wu, Xu Fang, Zhaoqiong Zhu

**Affiliations:** Department of Anesthesiology, Affiliated Hospital of Zunyi Medical University, Zunyi City, Guizhou Province, 563000, China; Department of Anesthesiology, Affiliated Hospital of Zunyi Medical University, No. 149 Dalian Road, Huichuan District, Zunyi City, Guizhou Province, 563000, China

**Keywords:** NUDT21, POCD, cognitive impairment, apoptosis, LIMK2

## Abstract

Postoperative cognitive dysfunction (POCD) is a common complication of cognitive decline after surgery and anesthesia. Sevoflurane, as a commonly used anesthetic, was found to cause POCD. Nudix Hydrolase 21 (NUDT21), a conserved splicing factor, has been reported to exert important functions in multiple diseases’ progression. In this study, the effect of NUDT21 on sevoflurane-induced POCD was elucidated. Results showed that NUDT21 was down-regulated in the hippocampal tissue of sevoflurane-induced rats. Morris water maze test results revealed that overexpression of NUDT21 improved sevoflurane-induced cognitive impairment. In addition, TUNEL assay results indicated that enhanced NUDT21 alleviated sevoflurane-induced apoptosis of hippocampal neurons. Furthermore, overexpression of NUDT21 suppressed the sevoflurane-induced LIMK2 expression. Taken together, NUDT21 alleviates sevoflurane-induced neurological damage in rats by down-regulating LIMK2, providing a novel target for the prevention of sevoflurane-induced POCD.

## 1 Introduction

Postoperative cognitive dysfunction (POCD) is a common complication with long-term consequences after surgery and anesthesia, characterized by cognitive decline [1,2,3]. Elderly patients are susceptible to suffering from POCD after surgery and anesthesia [4]. The incidence of POCD in elderly patients was 28.5% at 1 week after operation, and patients with POCD need a prolonged length of hospital stay and higher hospital costs [5]. POCD is proved to be associated with neurodegenerative diseases, such as Alzheimer’s disease in elderly patients [4,6]. Besides, patients with POCD showed significantly higher mortality than patients without POCD [7]. Therefore, POCD is a significant threat to public health, especially the health of elderly patients. Sevoflurane is a commonly used anesthetic in the clinic, and emerging evidence revealed that sevoflurane could induce POCD [8]. Hence, it is essential to investigate the mechanism of sevoflurane-induced neurotoxicity to prevent POCD.

Nudix Hydrolase 21 (NUDT21) is a member of the Nudix family of hydrolases and encodes Cleavage Factor Im 25 (CFIm25) [9]. It acts as a conserved splicing factor and is involved in eukaryotic pre-mRNA assembly due to possessing the cleavage factor Im (CFIm) complex [9,10]. Accumulating evidence revealed that NUDT21 participated in regulating many diseases [11,12,13]. For example, Weng et al. found that knockdown of NUDT21 amplified dermal fibrosis via alternative polyadenylation [11]. Zheng et al. reported that inhibition of NUDT21 suppressed proliferation and promoted apoptosis of pancreatic ductal adenocarcinoma via EIF2 signaling [12]. Sommerkamp et al. demonstrated that NUDT21 is involved in glutaminase isoform switching [13]. In addition, recent research revealed that NUDT21 participated in cognitive regulation [14]. Alcott et al. demonstrated that partial loss of NUDT21 caused learning deficits and aberrant neuronal alternative polyadenylation [14]. Furthermore, NUDT21 was proved to modulate the expression of LIMK2, a protein involved in neuronal growth and morphology, synaptic function, pain response, learning, and memory [9,15]. Therefore, we inferred that NUDT21 might be involved in sevoflurane-induced POCD.

In this study, the sevoflurane-induced rat model was constructed to investigate the effect of NUDT21 on neurological damage and to explore the potential mechanism. The findings revealed that NUDT21 improved sevoflurane-induced neurological damage via modulating LIMK2, providing a novel target for POCD prevention.

## 2 Materials and methods

### 2.1 Animals

Sprague-Dawley rats aged 15 months were acquired from Experimental Animal Center of Chongqing Medical University (Chongqing, China). All rats were allocated in the control, SEV, SEV + AAV-scramble, and SEV + AAV-NUDT21groups. There were nine rats in each group. The control group rats were exposed to mixed gas (1 L/min O_2_ + 1 L/min air), while the rats in the SEV group were exposed to 3.2% sevoflurane with a total flow of 1 L/min O_2_ and 1 L/min air for 2 h. The rats in the SEV + AAV-scramble group and the SEV + AAV-NUDT21group were first injected with 3.5 × 10^10^ drp AAV-scramble or AAV-NUDT21 (Hanbio, Shanghai, China) to one hippocampus (stratum lacunosum moleculare). These rats were exposed to 3.2% sevoflurane with a total flow of 1 L/min O_2_ and 1 L/min air 1 month later.


**Ethical approval:** The research related to animal use has been complied with all the relevant national regulations and institutional policies for the care and use of animals and has been aproved by the Ethics Committee of AffiliatedHospital of Zunyi Medical University (Approval No. KLL-2021-342).

### 2.2 Quantitative real-time polymerase chain reaction (qRT-PCR)

RNA sample isolation was conducted utilizing the PureLink™ RNA Mini Kit, and cDNA was generated using the DyNAmo cDNA Synthesis Kit (Thermo Fisher Scientific, Waltham, MA, USA). For NUDT21 mRNA detection, DyNAmo Flash SYBR Green qPCR Kit (Thermo Fisher Scientific, Waltham, MA, USA) was applied to qRT-PCRs on Light Cycler 480 II Real-Time PCR System (Roche Diagnostics, Basel, Switzerland) using GAPDH as the control. The primer sequences of NUDT21 [16] are presented in Table 1. The relative expression was calculated using the 2^−ΔΔCT^ method.

**Table 1:** The primer sequences used in qRT-PCR

Gene name	Forward	Reverse
*NUDT21*	5′-GTCAACCAGTTCGGCAACAAG-3′	5′-AGCTGTCCTTCTCATAGAGGG-3′

### 2.3 Western blot

Tissues were homogenized by radio-immunoprecipitation (RIPA) assay lysis buffer, and concentration determination was determined using the bicinchoninic acid (BCA) method. Tissue lysate was size-fractionated on sodium dodecyl sulfate polyacrylamide gel electrophoresis (SDS-PAGE) gels and transferred to PVDF membranes. The membranes were incubated with primary antibodies including NUDT21 (1:500), Bax (1:1,000), Bcl-2 (1:1,000), and GAPDH (1:2,500) antibodies (Abcam, Cambridge, MA, UK) overnight at 4°C after impeding non-specific binding, followed by treating with secondary antibody IgG H&L (HRP) (Abcam, Cambridge, MA, UK) at 37°C. After that, the blots were visualized utilizing the ECL detection system (Bio-Rad, Hercules, CA, USA).

### 2.4 Morris water maze test

Morris water maze test was used to evaluate the cognitive ability of rats [17]. The water maze consisted of a circular pool with 120 cm in diameter and filled with opaque water of 22°C. The maze was designated as four quadrants, and an escape platform was put in a quadrant at about 2 cm underwater. The rats were trained for 4 days with 4 trials each day, followed by a probe trial on the fifth day. During the training, rats were allowed to swim in the pool for the 120 s, and the time reached the platform was recorded. If the rats found the platform, the rat stayed on the platform for 10 s. If the rats did not find the platform, they were guided to the platform. During the probe trial, the rat was placed in the pool and swam for 60 s. The time crossing the platform and the time in the target quadrant were recorded. Besides, the swim routes of the rats were tracked automatically.

### 2.5 Terminal deoxynucleotidyl transferase dUTP nick end labeling (TUNEL)

For hippocampal tissue neurons apoptosis determination, TUNEL assay was conducted utilizing Click-iT Plus TUNEL Assay Kit (Invitrogen) in line with the supplier’s protocols. In short, tissue slides were fixed in 4% paraformaldehyde and permeabilized in proteinase K solution for 15 min, respectively. Then, the TdT reaction buffer was added to the slides for 10 min at 37°C, followed by incubating with the Click-iT™ Plus TUNEL reaction cocktail for 30 min at 37°C protected from light. Afterward, the cell nucleus was dyed with DAPI. The fluorescence microscope observed the staining results. The apoptosis proportion is the ratio of TUNEL-positive cells to the total cells.

### 2.6 Immunofluorescence

For immunofluorescence, tissues were immersed in 4% paraformaldehyde for fixation. After unmasking the epitope using 0.2% Triton X-100 and blocking nonspecific binding using 3% BSA, the tissues were probed with anti-LIMK2 antibody (1:200; Cell Signaling Technology) overnight at −4°C. Then, tissues were incubated with secondary antibody IgG H&L (HRP) (Alexa Fluor^®^ 594) (Abcam, Cambridge, MA, UK) for 1 h at room temperature. The cell nucleus was dyed with DAPI. The staining images were obtained under a confocal laser scanning microscopy.

### 2.7 Statistical analysis

Data were represented as mean ± standard deviation and statistically analyzed with SPSS 20.0 (IBM, SPSS, Chicago, IL, USA). Student’s *t*-test and one-way analysis of variance were typically used to test group difference. *P* < 0.05 was assumed to be statistically significant.

## 3 Results

### 3.1 NUDT21 was down-regulated in the hippocampal tissue of sevoflurane-induced rat

**Figure 1:**
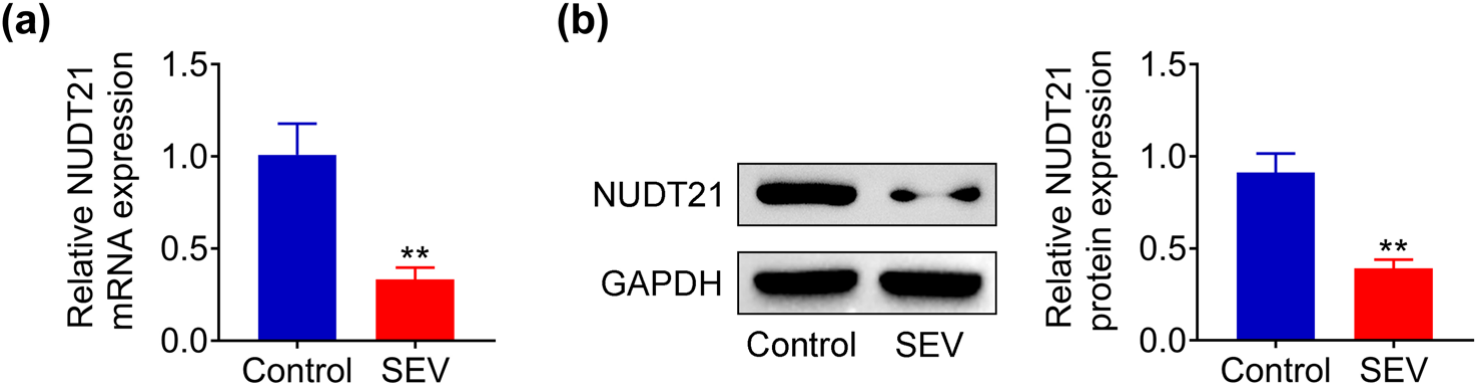
NUDT21 was down-regulated in the hippocampal tissue of sevoflurane-induced rats. (a) The NUDT21 mRNA in hippocampal tissue of sevoflurane-induced rats was determined using qRT-PCR. (b) The protein level of NUDT21 in hippocampal tissue of sevoflurane-induced rats was detected using western blot. **P < 0.01.

To investigate the action of NUDT21 on sevoflurane-induced COPD, the expression of NUDT21 in rats treated with 3% sevoflurane for 6 h was detected. NUDT21 mRNA was found to be decreased in hippocampal tissue of sevoflurane-induced rats (*P* < 0.01, Figure 1a). Similarly, Western blot results showed that the protein level of NUDT21 was reduced in hippocampal tissue of sevoflurane-induced rats (*P* < 0.01, Figure 1b). Therefore, NUDT21 was down-regulated in the hippocampal tissue of sevoflurane-induced rats.

### 3.2 NUDT21 improved sevoflurane-induced cognitive impairment in rats

**Figure 2:**
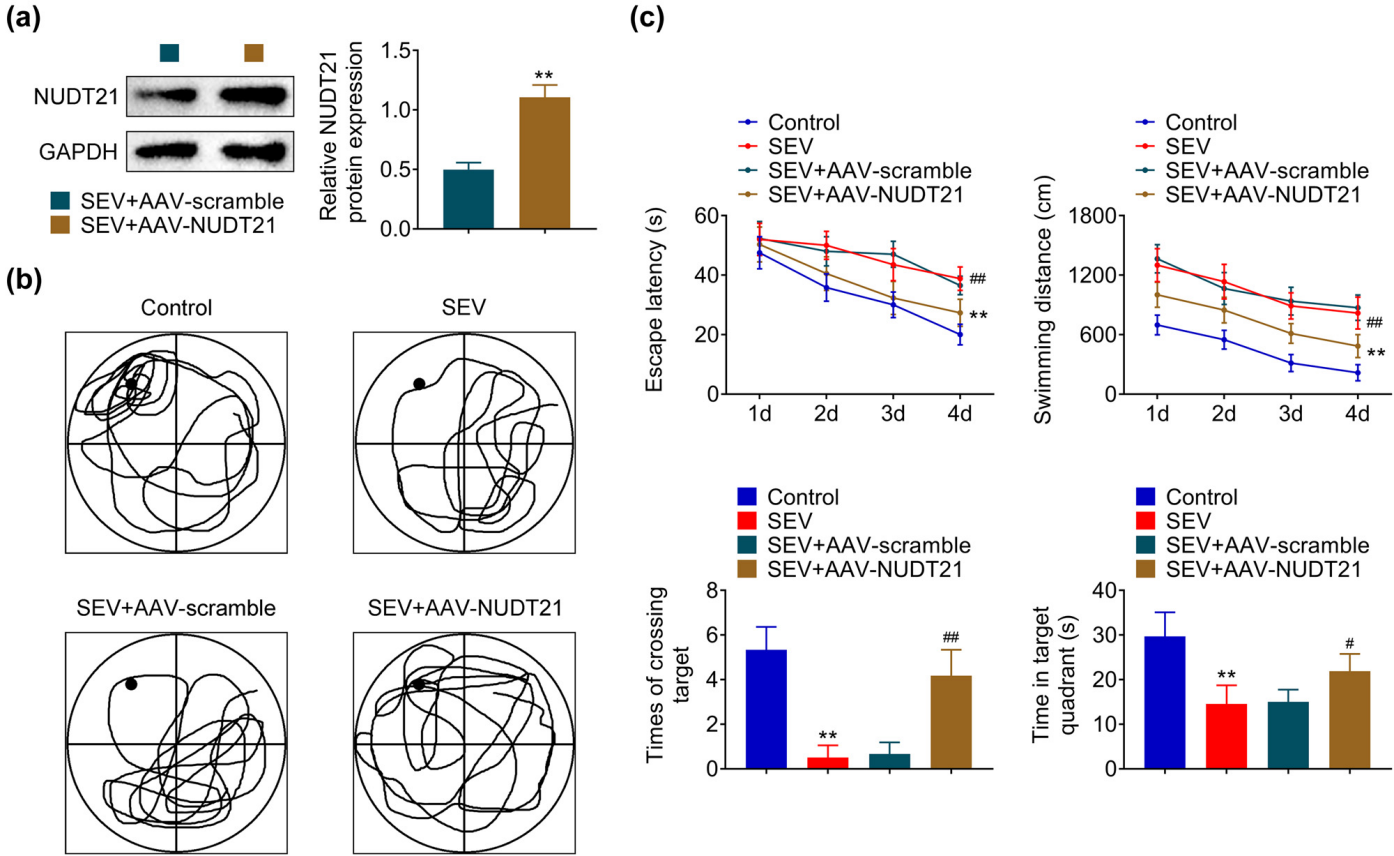
NUDT21 improved sevoflurane-induced cognitive impairment in rats. (a) The protein level of NUDT21 in hippocampal tissue of sevoflurane-induced rats after transfected with AAV-NUDT21 was detected by western blot. (b) The movement route of sevoflurane-induced rats after transfected with AAV-NUDT21 was recorded during the Morris water maze test. (c) The escape latency, swimming distance, times of crossing target, and time in the target quadrant of sevoflurane-induced rats after transfected with AAV-NUDT21 were measured during the Morris water maze test. **P < 0.01. ^#^ *P* < 0.05. ^##^ *P* < 0.01.

To gain insight into the effect of NUDT21 on sevoflurane-induced neurological damage, the cognitive impairment of sevoflurane-induced rats was assessed after transfected with AAV-NUDT21. AAV-NUDT21 significantly enhanced the NUDT21 level (*P* < 0.01, Figure 2a). Morris water maze test was then conducted to evaluate the cognitive impairment of rats. The movement route of rats during the Morris water maze test is presented in Figure 2b. Results showed that the escape latency and swimming distance were increased in sevoflurane-induced rats, and enforced NUDT21 reversed the phenomenon (*P* < 0.01, Figure 2c). In addition, overexpressed NUDT21 increased the times of crossing target and time in the target quadrant of sevoflurane-induced rats (*P* < 0.01, Figure 2c). Taken together, NUDT21 improved sevoflurane-induced cognitive impairment in rats.

### 3.3 NUDT21 improved sevoflurane-induced apoptosis of hippocampal neurons

**Figure 3:**
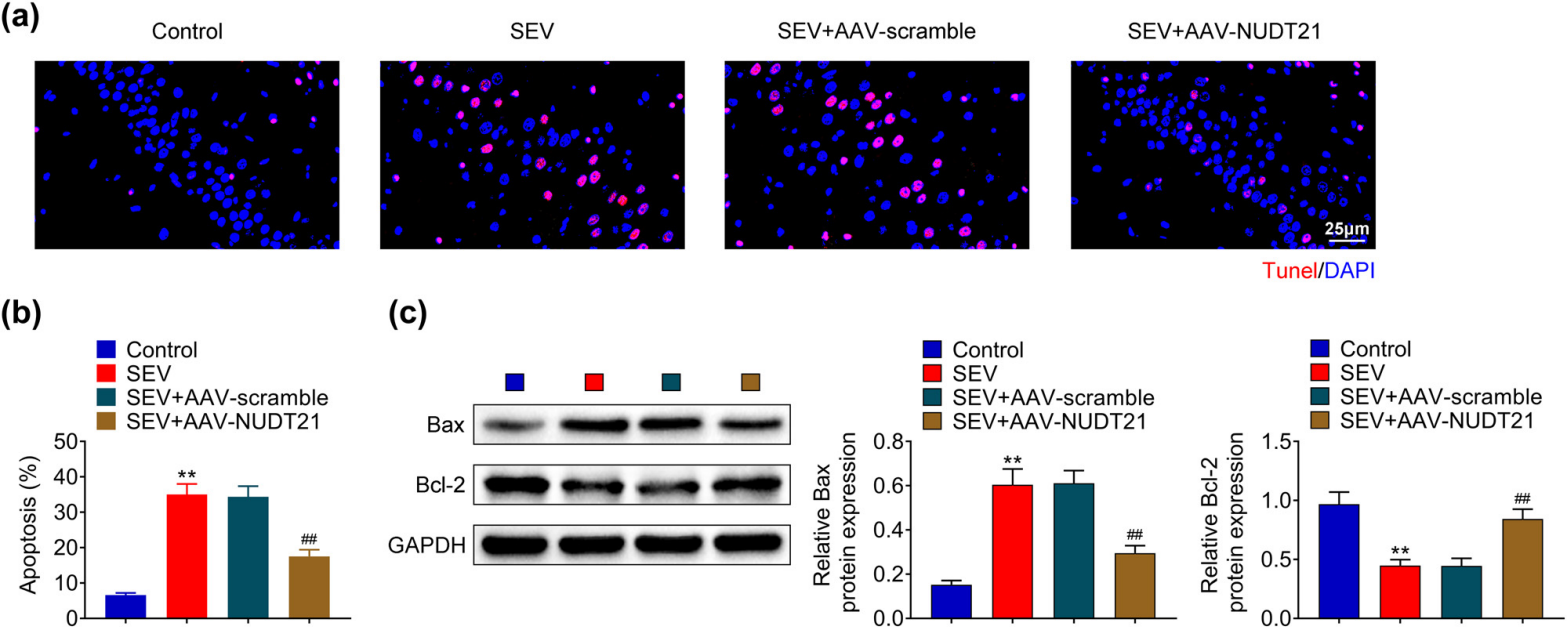
NUDT21 ameliorated sevoflurane-induced neurons apoptosis in hippocampal tissue. (a) Hippocampal neurons apoptosis of sevoflurane-induced rats after transfected with AAV-NUDT21 was evaluated using TUNEL assay. (b) Quantitative results of apoptosis rate in hippocampal neurons of sevoflurane-induced rats after transfected with AAV-NUDT21. (c) The protein levels of Bax and Bcl-2 in hippocampal neurons of sevoflurane-induced rats after transfected with AAV-NUDT21 were detected using Western blot. **P < 0.01. ^##^ *P* < 0.01.

Next, the role of NUDT21 in hippocampal neurons apoptosis of sevoflurane-induced rats was explored. TUNEL staining results showed that overexpression of NUDT21 suppressed sevoflurane-induced apoptosis of hippocampal neurons (*P* < 0.01, Figure 3a and b). The apoptosis-related protein Bax was increased, and Bcl-2 was decreased in hippocampal of sevoflurane-induced rats, which were reversed by overexpression of NUDT21 (*P* < 0.01, Figure 3c). Thus, the findings indicated that NUDT21 improved sevoflurane-induced apoptosis of hippocampal neurons.

### 3.4 NUDT21 down-regulated the expression of LIMK2

**Figure 4:**
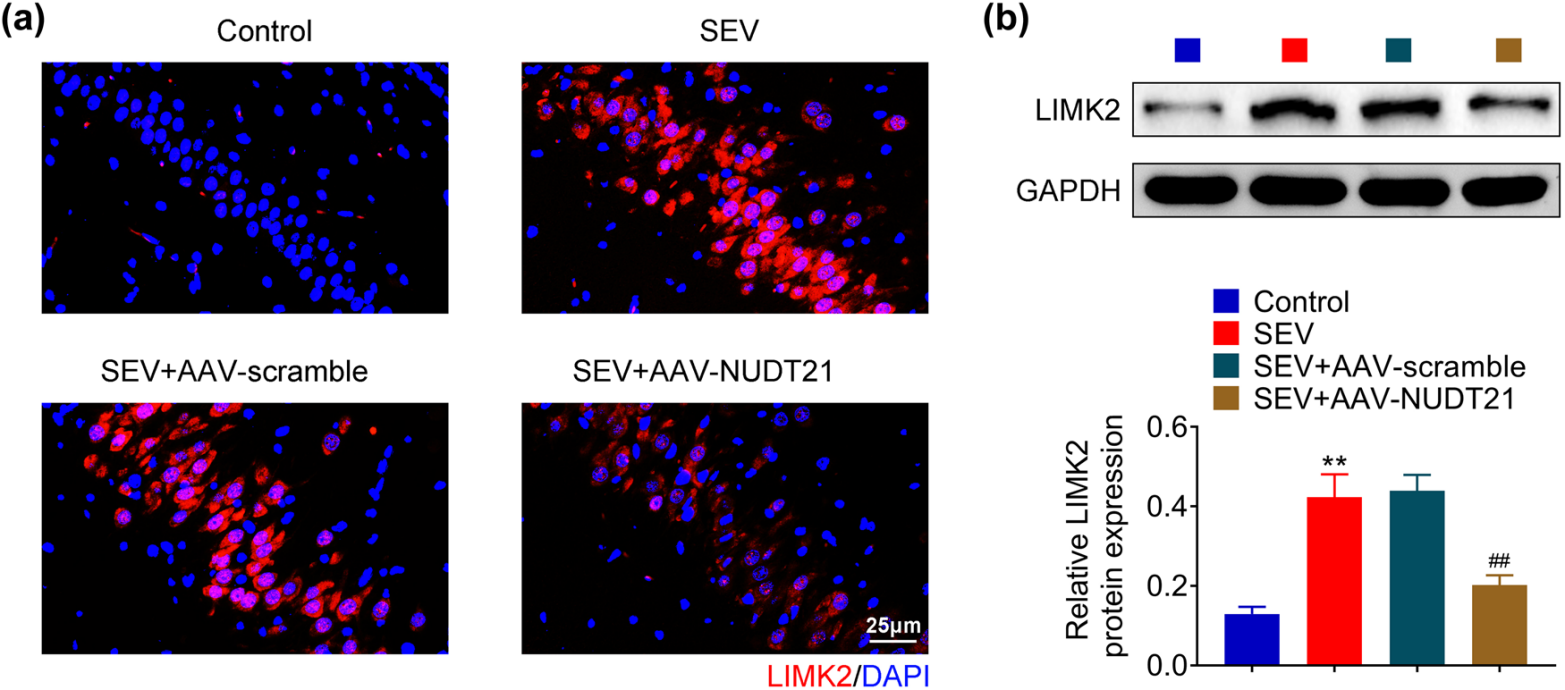
NUDT21 down-regulates LIMK2 expression. (a) The LIMK2 expression in hippocampal tissue of sevoflurane-induced rat after transfected with AAV-NUDT21 was assessed using Immunofluorescence. (b) The LIMK2 expression in hippocampal tissue of sevoflurane-induced rat after transfected with AAV-NUDT21 was assessed by Western blot. **P < 0.01. ^##^ *P* < 0.01.

To clarify the possible mechanism of NUDT21 on sevoflurane-induced POCD, the expression of LIMK2 in the sevoflurane-induced rat after transfected with AAV-NUDT21 was assessed. Immunofluorescence staining results showed that sevoflurane treatment increased the expression of LIMK2 in hippocampal tissue of rats and it was inhibited by enforced NUDT21 (Figure 4a). Similarly, Western blot results also proved that overexpression of NUDT21 suppressed sevoflurane-induced LIMK2 level in hippocampal tissue of rats (*P* < 0.01, Figure 4b). Hence, NUDT21 decreased the expression of LIMK2 in sevoflurane-induced rats.

## 4 Discussion

In recent years, there is a growing concern that anesthetic can have toxic effects on neurons and cause persistent cognitive damage [18]. As a commonly used anesthetic, sevoflurane was found to cause POCD [19]. However, the exact mechanism of sevoflurane caused POCD remained to be further elucidated. NUDT21, a conserved splicing factor, was revealed to play important roles in multiple diseases’ progression, while its influence on sevoflurane-induced POCD remains unclear.

Therefore, this study investigated the action of NUDT21 on sevoflurane-induced neurological damage. The expression of NUDT21 in sevoflurane-induced rats was determined. The results revealed that NUDT21 was down-regulated in the hippocampal tissue of sevoflurane-induced rats. The decreased expression of NUDT21 was also found in cancers, such as hepatocellular carcinoma, breast cancer, and bladder cancer [9,10,20]. However, up to now, the current study is the first to report the aberrant expression of NUDT21 in the hippocampal tissue of sevoflurane-induced rats. Previous study reported that NUDT21 was a highly constrained gene and it was closely related to health as its 125 missense and 13 loss-of-function variants would be expected in NUDT21 if loss-of-function variants were not pathogenic, but instead there were only 15 missense and 0 loss-of-function variants [21]. Hence, the aberrant expression of NUDT21 in the hippocampal tissue of sevoflurane-induced rats suggested that it might participate in the POCD development.

POCD is mainly manifested as the decline in cognitive function after surgery and anesthesia [22]. The previous study revealed that the cognitive function of sevoflurane anesthesia patients after operation was significantly worse than pre-operation [23]. Therefore, to clarify the role of abnormally expressed NUDT21 in sevoflurane-induced neurological damage, the cognitive impairment was detected using the Morris water maze test. The Morris water maze test is a well-established test for assessing spatial learning and memory [24]. The advantage of the test is that it can be carried out quickly without the restriction of water and food [25]. Furthermore, the factors affecting spatial learning and memory ability can be overcome by adjusting the test method [25]. According to the results of Morris water maze test, we found that sevoflurane exposure inhibited the spatial learning ability, as reflected by the index of escape latencies and spatial memory reflected by the time spent in the target quadrant. In other words, sevoflurane treatment suppressed the cognitive function of rats, which was consistent with the previous studies [26,27]. However, the cognitive impairment induced by sevoflurane was alleviated by overexpressed NUDT21, suggesting that NUDT21 protected against sevoflurane-caused cognitive impairment. Similarly, Alcott et al. reported that partial loss of NUDT21 caused learning deficits and cortical hyperexcitability via regulating alternative polyadenylation and protein levels in hundreds of genes [14].

Increasing studies found that neuronal apoptosis was a critical cause of POCD development [28]. For example, inhibition of α-Synuclein accumulation alleviated neuronal apoptosis and improved POCD in aged mice [29]. Besides, sevoflurane was demonstrated to induce apoptosis of the nervous system, which was closely associated with the POCD occurrence [30]. Ling et al. also found that sevoflurane induced neuronal apoptosis via enhancing DNMT3L expression and promoting methylation of PSD95 promoter in POCD [31]. Thus, the current study investigated neuronal apoptosis using TUNEL assay to explain the role of NUDT21 in sevoflurane-induced POCD. As expected, sevoflurane treatment induced apoptosis of hippocampal neurons. Nevertheless, enhanced NUDT21 suppressed sevoflurane-induced hippocampal neuronal apoptosis. In the study performed by Brumbaugh et al., NUDT21 was considered a novel cell fate regulator by connecting alternative polyadenylation [16]. Zheng et al. revealed that knockdown of NUDT21 promoted apoptosis of pancreatic ductal adenocarcinoma [12]. To our knowledge, the present study is the first to reveal the apoptosis inhibitory function of NUDT21 on sevoflurane-induced POCD. Based on the above evidence, we inferred that sevoflurane treatment suppressed the expression of NUDT21 to promote the hippocampal neuronal apoptosis, thereby inducing POCD. Therefore, restoring the expression level of NUDT1 may be an effective way to prevent sevoflurane-induced POCD.

LIMK2 was a member of the LIM kinase (LIMK) family of serine/threonine kinases [32]. Recent studies validated that LIMK2 regulated neuronal growth and morphology, synaptic function, pain response, neuronal apoptosis, learning, and memory [15,33]. In this study, the expression of LIMK2 was explored to explore the precise regulatory mechanism of NUDT21 on sevoflurane-induced POCD. The results indicated that sevoflurane stimulation increased the LIMK2 expression, which was inhibited by NUDT21. These findings were consistent with the study performed by Xiong et al. [9]. They found that LIMK2 was downregulated in NUDT21 overexpressed cells but was upregulated in NUDT21-knockdown cells [9]. As a conserved splicing factor, NUDT21 encoded CFIm25 which binds UGUA sequences in pre-mRNA and the CFIm complex recruits the enzymes essential for cleavage and polyadenylation [21]. Since the UGUA binding sites are usually enriched at the distal polyadenylation sites, proximal cleavage sites were more frequently used after NUDT21 expression was reduced [21]. Therefore, downregulation of NUDT21 caused 3′UTR shortening in hundreds of genes, thereby increasing protein levels of a subset of those genes [21]. In this study, the expression of LIMK2 was negatively regulated by NUDT21. This finding indicated that NUDT21 might modulate LIMK2 expression via alternative polyadenylation. Together, NUDT21 might attenuate sevoflurane-induced neurological damage in rats by down-regulating LIMK2.

In conclusion, NUDT21 was down-regulated in hippocampal tissue of sevoflurane-induced rats. Overexpression of NUDT21 alleviated sevoflurane-induced neurological damage by down-regulating LIMK2. These findings provided a novel target for the prevention of sevoflurane-induced POCD.
